# Decision making: rational or hedonic?

**DOI:** 10.1186/1744-9081-3-45

**Published:** 2007-09-11

**Authors:** Michel Cabanac, Marie-Claude Bonniot-Cabanac

**Affiliations:** 1 Department of Physiology, Faculty of Medicine, Laval University, Canada

## Abstract

Three experiments studied the hedonicity of decision making. Participants rated their pleasure/displeasure while reading item-sentences describing political and social problems followed by different decisions (Questionnaire 1). Questionnaire 2 was multiple-choice, grouping the items from Questionnaire 1. In Experiment 1, participants answered Questionnaire 2 rapidly or slowly. Both groups selected what they had rated as pleasant, but the 'leisurely' group maximized pleasure less. In Experiment 2, participants selected the most rational responses. The selected behaviors were pleasant but less than spontaneous behaviors. In Experiment 3, Questionnaire 2 was presented once with items grouped by theme, and once with items shuffled. Participants maximized the pleasure of their decisions, but the items selected on Questionnaires 2 were different when presented in different order. All groups maximized pleasure equally in their decisions.

These results support that decisions are made predominantly in the hedonic dimension of consciousness.

## Background

"Gut reaction" is efficacious" [1]

For several decades, research in judgment and decision making has examined behavioral violations of rational choice theory [2,3]. For example, Baron showed convincingly that many decisions appear to be irrational, as if decision-makers were indifferent to the consequences of their decisions [2]. Erev and Roth showed that decisions in gambling situations are made at low rationality, the gamblers' aim being to maximize reinforcement [4,5]. Berridge concluded that a rational decision is a decision that maximizes utility (with all the ambiguity contained in the word utility) [6].

Epstein's [7] proposal of a "dual-process" in decision making casts some light on that experiential" rational, abstract, and analytical treatment of the available information, and a second one, "experiential" and "emotionally driven". According to Epstein, both systems fulfill different functions. Loewenstein and co-workers [8] proposed also an alternative theoretical perspective, the risk-as-feelings hypothesis, that highlights the role of affect experienced at the moment of decision making. Similar views were expressed by Reyna & Farley [9]: "Risky decisions making can be roughly divided into a) those [...] that adhere to a rational behavioral decision-making framework [...] and b) those that emphasize non-deliberative reaction to the perceived gists or prototypes in the immediate decision environment. " The experiential system is present in animals and leads to effortless decisions. The analytical system emerged more recently in humans with the development of language. The present experiments were developed in the same framework, exploring hedonicity pitted against several variables involved in decision making: time available for decision making, rationality, and recognition.

Maximization of hedonic experience is a universal mechanism inherited by humans to motivate behavior [10] and makes pre-rational decisions [11-13]. Emotion interferes powerfully with decision making [14]. Mellers recently proposed an account of emotional experiences associated with the outcomes of decisions called "decision affect theory." It incorporates utilities, expectations, and counterfactual comparisons into hedonic responses. That is, people choose the risky options for which they expect to feel better on average [15,16]. Conversely, positive moods may increase sensitivity to the meaning-relevance of a situation [17]. Price et al. also have proposed a commonality between cognitive processes underlying emotions and choice [18]. Such views are similar to Cabanac's notion of pleasure being the "common currency," if one accepts that emotion is basically an intense hedonic experience [19]. Slovic and co-workers reached a similar conclusion, using the terms affectivity and affect to qualify that something is good or bad [20].

Optimization of everyday life decisions is similar in perception and memory processes [21], which suggests that the laws of mental optimization are similar and possibly universal. In previous experiments we have studied the place of pleasure in decisions made in various domains [22,23], yet, it was noticeable that never, in any of our previous studies where we explored decisions in various fields in relation to hedonic experience did any participant choose 100% responses providing pleasure. Thus, other factors must enter, of course, into account besides hedonicity. A recent review suggested that both rational and intuitive decision making processes are likely to play an important role in ethical decision making [24]. "A vast area exists between irrational and rational that can be called *arational*" [25]. In the present paper we examined the hypothesis that such an arational process is actually hedonic. We studied the influence of hedonicity in three separate experiments. The aim of the present work was double: first, to verify whether previous experimental data showing the preeminent role of pleasure in decision making, could be confirmed while the previous experimental protocols were modified, and second, an attempt to falsify our working paradigm by removing the tautology involved in studies where participants rate hedonically various items, then select those they prefer. Both aims were tackled in the following experiments.

## Methods

### Participants

One hundred and twenty persons volunteered to participate anonymously in the study. They were recruited at random on campus and in supermarkets. The only criterion for selection of volunteers was a progressive attempt to match, as well as possible, sex and age ratios in Experiment 1, and then less so in Experiments 2, and 3. Each person participated individually in a private interview. The duration for answering Questionnaires 1 and 2 (below) were timed. The only personal data recorded were participants' age and sex. Laval University Committee for the Ethics of Research approved the study.

### Questionnaires

The general principle consisted in presenting two questionnaires dealing with political and social problems. Because Grammar, Mathematics, Aggressiveness and Ethics had been studied in our previous studies, we explored here a new field, politics. There were 10 general topics: Irak war, Globalisation, Immigration, Family, Homosexuality, Abortion, Genetically Modified Organisms (GM Foods), North Korea, Palestine and Israel, and Cuba (see Additional file 1). Each of the general themes was presented five times in a randomized order with each time a different solution to the political/social problem, which resulted in 50 items. The five possible solutions offered on each theme tried to cover a broad spectrum of decisions from staid conservative to extreme liberal.

In Questionnaire 1, the 50 items were presented one after the other. The participants were invited to read carefully the first item, then write down on an answer sheet the amount of pleasure or displeasure evoked in them by that item; pleasure and displeasure were considered as belonging to the same dimension [11]. The quantitative rating would be positive for pleasure, negative for displeasure, and zero for indifference (or for "I don't know"). The amplitude of the scale was left to the participants in order to let them use a scale with which they would feel at ease. Some chose from -5 to +5, others -10 to +10, or -20 to +20, etc. Such a liberty was given because results would be compared within each participant's results.

In Questionnaire 2, the 50 items of Questionnaire 1 were presented grouped by five items on the same theme (see Additional file 1). This resulted in a multiple-choice Examination type with 10 entries, each containing all the 5 items on the same theme. The participants read the 5 items of the given entry and wrote on a new answer sheet which political solution they would decide to choose, in case were they in an executive position allowing decision to be adopted. Any mention of hedonicity was carefully avoided. Then the participants went to entry 2, then 3, up to the 10th and made the same decisions.

### Data analysis

Once the data had been collected for all participants, the ratings on Questionnaire 1 and decisions on Questionnaire 2 were compared as follows: because all participants adopted different ranges of ratings for Questionnaire 1, we ranked them according to hedonic ratings rather than comparing the absolute ratings given to the 5 items of each theme. For each theme the lowest hedonic rating was given rank 1, and the highest hedonic rating was given rank five. Thus for each participant the total rating for the 10 entries could extend from 10, minimum, to 50 maximum possible. A total rating of 10 would mean that the participant would have selected systematically in Questionnaire 2 the ten items most disliked, or least liked. A total rating of 50 would mean that the participant would have selected systematically the ten items that he/she liked most, or disliked least. A total rating of 30 would mean that the participant was likely to have selected at random the items, as 30 is chance.

## Experiment 1: Speed

"There are many different relationships and interactions between time and decision making, and no single summary can do justice to this topic" [26]. In Experiment 1, the duration allotted to answer Questionnaire 2 was manipulated. The aim was to verify whether hedonicity would be the deciding variable when people are in a hurry to decide while they would be more rational when they have the time.

### Methods

In that study 60 participants, 30 men (m ± se, 51.6 ± 2.7 yr) and 30 women (50.8 ± 2.2) were presented with the two questionnaires, as described above. The mean ages were similar enough to be insignificant (Student's t = 0.2, D.F. 58, P = 0.82). After reading and rating the 50 items of Questionnaire 1 the participants were divided into two groups before answering Questionnaire 2. The first group, 15 men (m ± se, 55.4 ± 3.5 yr) and 15 women (m ± se, 47.1 ± 3.5 yr) was casually instructed to take their time in answering Questionnaire 2 without giving them any indication that duration of response was measured. As a result they completed that Questionnaire in a m ± se, 6.0 ± 0.4 min.

The second group of 30 participants, contained also 15 men (m ± se, 47.9 ± 4.1 yr) and 15 women (m ± se, 54.6 ± 2.5 yr). They were instructed to read the five topics of each entry once and to decide quickly which political decision they would make. As a result they completed that Questionnaire in a m ± se, 3.8 ± 0.2 min. The duration of both groups thus differed by 2.2 min (Student's t = 5.0, D.F 58, P < 0.0001).

### Results and discussion

There was no influence of age (correlation 0.135, Z-value 1.025, P > 0.3) nor of sex on the decisions made on Questionnaire 2 (Fischer's PLSD, P = 0.91). Therefore no consideration of this factor will be given in the following nor in Experiment 2. The mean scores of selected items in Questionnaire 2 (known from ratings of items in Questionnaire 1) was 41.4, *i.e*. way above chance: 30 (One-sample *t *test = 76.1, D.F. 59, P < 0.0001).

**Figure 1:**
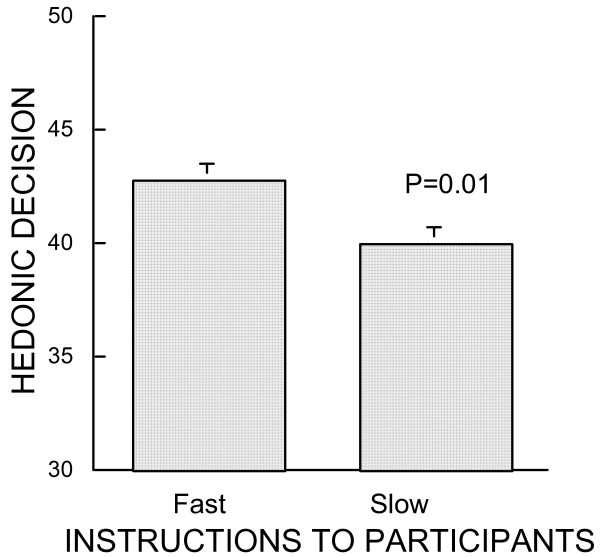
Mean results of Experiment 1. Participants rated 50 items as more or less pleasant to read (Questionnaire 1), then selected the ones they would decide to do (Questionnaire 2). Ordinates indicate the number of times participants chose in Questionnaire 2 the most positively hedonic; minimum 30 is chance, maximum 50 is systematic decision in favor of most pleasant items. Fast column gives the mean results of 30 participants who were instructed to decide quickly on Questionnaire 2. Control column gives the mean results of 30 participants who received instruction to take their time regarding decision on Questionnaire 2.

The above analysis of Questionnaire 2 showed that all participants had selected the items that they had described as pleasant in Questionnaire 1. Yet the results of both groups were different: the duration given to the participants to make a decision on Questionnaire 2 was highly significantly influential (Fig. 1). The mean ranking of participants who had time to decide (40.0 ± 0.7) was lower than that of participants who were instructed to hurry to decide (42.8 ± 0.7), (Fischer's PLSD, P = 0.011).

Ariely & Zakay [26] made an important contribution when they showed that the time available for decision is an important parameter in decision making. This was confirmed by Diederich [27]. The results of Experiment 1 result support their contention that the time allowed to decide takes place in the process of decision making. When time is scarce, decision-makers trust their hedonic experience. Such a difference might be related to the fact that different brain structures are activated when decision is urgent or may wait [28].

Even when they had to examine quickly the five items of all 10 decisions to be made, the participants never selected systematically the items which they had rated highest on Questionnaire 1. Their decisions tended to be positive and hedonically much higher than chance (41.4 *vs*. 30) but they never reached the maximal possible rating of 50. Such a difference between actual decision from maximal possible pleasure leads one to suspect that other factors than pleasure enter into play when a participant decides. Such a factor could be rationality. When there is no time pressure, the duration taken to make a decision is a function of the strength of the conflict between several possible solutions [27]. This was confirmed in the group of participants who had time to weigh their decisions, as the hedonicity of their final choices were lower than those of control participants. One may suspect that rationality entered into their decisions.

## Experiment 2: Rationality

In Experiment 2, the participants were instructed to select most rational issues in Questionnaire 2.

### Methods

In that study, 60 participants, 37 men (m ± se, 54.9 ± 1.6 yr) and 23 women (51.0 ± 1.5), were presented with the same two questionnaires, as described above. The mean ages were similar enough to be insignificantly different (Student's *t *= 1.4, D.F. 58, P = 0.16). After reading and rating the 50 items of Questionnaire 1, the participants were divided into two groups before answering Questionnaire 2. The first group (control n = 33, 21 men and 12 women) was asked to answer Questionnaire 2 without being instructed about rationality; thus, they received the same instructions as the control group in Experiment 1. The second group (n = 27, 16 men and 11 women) was instructed to answer rationally to Questionnaire 2 and to decide in favor of the political decisions that appeared most rational to them. All participants knew that they could ask questions about the Methods; none of them asked any question about the word 'rational,' thus indicating that they understood the word without ambiguity.

### Results and discussion

Again as in Experiment 1, in Experiment 2 there was no difference between the results obtained from men and from women (Fischer's PLSD, P > 0.13). There was no influence of age (correlation 0.201, Z-value 1.541, P > 0.12) on the results from Questionnaire 2.

**Figure 2:**
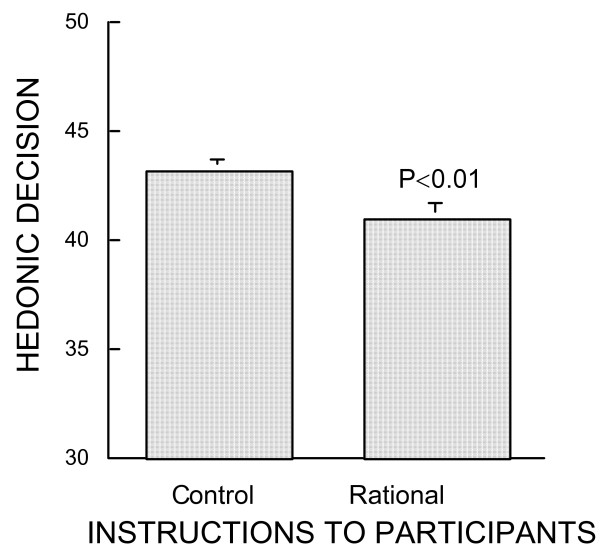
Mean results of Experiment 2. Same protocol as in Experiment 1 but the experimental group were instructed to select the most rational responses on Questionnaire 2. Ordinates indicate the number of times participants chose in Questionnaire 2 the most positively hedonic; minimum 30 is chance, maximum 50 is systematic decision in favor of most pleasant items. Rational column gives the mean results of 27 participants who were instructed to select the rational responses on Questionnaire 2. Control column gives the mean results of 33 control participants who received no instruction regarding rationality of decision on Questionnaire 2.

On the other hand, the rationality involved in making a decision on Questionnaire 2 was highly significantly influential (Fig. 2): the mean hedonic ranking of items selected by participants from the control group was (43.2 ± 0.7), *i.e*. 2.2 higher than that of participants who decided rationally (41.0 ± 0.5, Fischer's PLSD, P = 0.0025). Still, Fig. 2 shows that the items selected on a rational basis remained quite indicative of pleasure choice, as the mean ranking of rational decisions was more than 10 higher than chance (30). Such a correlation of hedonicity with rationality supports the contention that rational decisions provide pleasant experiences [29] even if less pleasant than those of spontaneous decisions. It follows that pleasure remains at the root of decision making.

## Experiment 3: Recognition heuristic

Gigerenzer and Todd [30] developed the *fast-and-frugal *approach to modeling human decision making; they challenged the rational approach and argued that because human decision-making process evolved in competitive environments, they needed to be fast, and because they evolved in changeable environments they needed to have the robustness that comes from simplicity. Maximization of hedonic experience fulfils both tasks. But, more specifically, Goldstein and Gigerenzer [31] considered recognition as a way to be successful in repeated circumstances. To explore the hypothesis of the previously evidenced role of pleasure in decision making might depend on a simple recognition of items by the participants, in Experiment 3, we presented the same items twice in order to verify whether participants would select behaviors that they had previously preferred.

### Methods

In that study 35 participants (21 men, m ± se: 51.6 ± 2.1 yr, and 14 women, 54.4 ± 1.3 yr. Fisher's PLSD, mean diff. 3.77, Crit. Diff. 4.8, P = 1.2), were presented with the two questionnaires, as described above. However, in that experiment, a different Questionnaire was presented (Questionnaire 3). In it, all the items of Questionnaire 1 were arranged not by theme, as in Questionnaire 2, but on the contrary were shuffled so that in each of all 10 entries on that questionnaire the 5 items were mixed in order not to have two items on the same problem (say, Irak, or abortion) twice in the same entry. Thus, all items on the 10 entries were on different themes. E.g. instead of presenting an entry with 5 different behaviors regarding, say, abortion, the entry would contain one item on abortion, one on immigration, one on Irak war, one on Cuba, and one on GM food. After that additional Questionnaire 3, the participant would return to the normal protocol, read Questionnaire 2 with again the same items but this time with all items grouped by theme, and decide which to choose. The intention with the protocol of Experiment 3 was to familiarize the participants with the various decisions and check whether recognition would influence their decisions. If that is the case one would expect participants to choose the same items in Questionnaires 3 and 2. What was counted for each participant, therefore, was the number of coincidences when a given item had been selected on both Questionnaires 3 and 2. The maximal number would be 10 coincidences, as there were 10 entries on both questionnaires.

### Results and discussion

In men and women the results were identical and superimposed therefore with those of the whole group: the mean (± se) number of coincidences was: 5.1 ± 0.2, out of a maximum possible 10, i.e. -4.8 lower than maximum (Student's *t*, 30.6, D.F. 34, P < 0.0001). Thus, the participants chose predominantly the items that they had rated as pleasurable, but recognition played little or no role in their decisions, as the items selected five times out of ten were not the same as those they had decided on Questionnaire 2.

Thus, if recognition influenced the participants' decisions as predicted from Goldstein and Gigerenzer [31], that influence was minor. Such a conclusion would confirm the fact that a long delay of 77 +3 d placed between Questionnaires 1 and 2 produced the same results as the above Experiments 1–3 (Bonniot-Cabanac & Cabanac, submitted). Yet in that experiment, too, participants maximized hedonicity in their decisions (m ± se) both in Questionnaires 2 and 3, with identical scores (43.1 ± 0.5) and (43.2 ± 0.5). Such a result also confirms that participants tended to maximize hedonicity, irrespective of the form of presentation of the various problems where they had to make decisions.

## General discussion

The aim of the paper was to address the interplay between hedonicity and choice. Specifically, we tested the hypothesis that the amount of subjective pleasure associated with a behavioral alternative affects that likelihood that this alternative will be chosen by a decision-maker. In order to test this hypothesis, the participants first assessed the pleasure associated with a range of choice alternatives (Questionnaire 1). Then they chose (Questionnaire 2). The results showed that participants significantly chose options that they had rated as pleasant, although they didn't choose always the most pleasant ones, and sometimes chose clearly unpleasant ones.

A criticism raised sometimes by anonymous reviewers of previous experiments has been that the approach with two questionnaires may suffer from some tautology, as one may expect the participants to select in Questionnaire 2 what they like. However the present work responds to that criticism and removes the tautology, as the results confirmed that the link between pleasure and decision did not follow automatically the trend to maximize pleasure. In none of the three experiments did any participant reach the total maximum of 50 that would mean selecting systematically the most pleasurable items. The choosing of pleasant items in Questionnaire 2, therefore, was not that obvious and not as tautological as one might have feared. Furthermore, the duration of the time available for decision making made a difference on their final choice: when participants had time to weigh their decisions, they selected pleasant responses, but less so than when they had to rush to decide. The same pattern took place when participants had to decide in favor of rational solutions. The pleasure ratings of decisions actually made remained vastly higher than chance, but nevertheless significantly less pleasant than when participants had to decide without focusing on rationality. Thus, the results failed to falsify our working paradigm and confirmed pour previous experimental data showing the preeminent role of pleasure in decision making [22,32] although the previous experimental protocols were modified.

Concepts of motivation are vital to progress in behavioral neuroscience [33]. Our present results obtained both in Experiment 1 and in Experiment 2 confirm that the pleasure experienced when the participants read different solutions to political problems correlated with their subsequent decisions on these items. Their pleasure/displeasure seemed to indicate the right solution whenever they made a political/social decision and seemed to provide decision-making clues. The present results thus confirm the hypothesis that maximization of experienced pleasure (i.e., experience value) and its counterpart, minimization of displeasure, occur in the decision-making process. This confirms several results obtained in previous experiments studying the role of pleasure on decisions in conflicts of motivations for physiological behaviors [34], and in various purely psychological fields: grammar, gambling, mathematics, politics, poetry, aggression, etc. [19,22,32,35] If we accept that emotion is "any mental experience with high intensity and high hedonicity", then the present results confirm the fundamental role of emotion in decision making [36] and that emotional intelligence predicts success in important domains, among them personal and work relationship [37,38]. A recent study showed that in absence of contextual cues or situational constraints, choices followed a pleasure-maximizing principle [39], a result closely similar to the present ones.

There was no difference in decisions made by participants whatever sex or age. The tendency to seek pleasure and avoid displeasure thus appears to be deeply rooted in human nature, as it covers a very broad area of the human mind and is independent of decision-makers' sex and age. This does not disprove Flynn, Slovic, & Mertz' [40] results that demonstrate socio-ethnical influence in the solution chosen to environmental risk, but shows that when a difference shows, it is more likely to be rational than hedonic. Such a profound influence of hedonicity is likely to be due to the antiquity of this mechanism, which is present in animals, too [41].

The fact that the time allowed for decision was an influential variable (Experiment 1) also suggests that hedonicity is the fundamental mechanism that takes place in emergency when there is no time for deep evaluation. Slovic and collaborators described recent empirical research illuminating "the affect heuristic" wherein people rapidly consult their affective feelings when making judgments and decisions. This heuristic enables us to be rational actors in many situations. It works beautifully when experience enables us to anticipate accurately how we will like or dislike the consequences of our decisions. However, it fails miserably when the consequences turn out to be much different than we anticipated. In the latter circumstances, the rational actor may well become the rational fool [42]. Recent microeconomics studies of consumer behavior showed that the price is traded in the buyers' mind against other pieces of information such as GMFoods influence on health, [43], or taste qualities of drinks [44], and that the hedonic experience has a major impact on final decision [45], a result confirmed by the present paper.

"The sentiment of mathematical elegance is nothing but the satisfaction due to some conformity between the solution we wish to discover and the necessity of our mind [...] This aesthetic satisfaction is consequently connected with the economy of thought" [46]. The hypothesis that hedonicity is the main key in decision making [11,47] has gained momentum in the recent years. Johnston [48] also reached the conclusion that hedonicity is the dimension of the mind that allows us to decide independently from rationality. Thus hedonicity is likely to explain intuition as a valuable and reliable problem-solving tool [49,50]. Decision-making seems to reflect two processes: a rational one, when the subject has the necessary information and cultural background, and a hedonic one, when the subject cannot think through the situation for time pressure or lack of information. The hedonic mechanism being more archaic, for obvious reasons rooted in physiology, zoology, and anthropology [51,52], would be common to all humans and would come into play when there is no time to be rational or when rationality fails to provide a solution [11,42]. A similar conclusion was also reached by Slovic and co-workers [12,20,42] who coined the term "affect heuristic" for that process. The results are consistent with the claim that non-rational processes contribute to decision making. Another support to the hypothesis – this time behavioral – can be found in the fact that pathological symptoms of stress occur when the algebraic sum of effort and reward is chronically negative [53].

Hedonicity was shown also to influence decision indirectly through the decision-makers' mood. Isen et al. have repeatedly shown that not only was decision easier when the decision-makers' mood was positive [54], but further that the quality of their decisions was also improved [55,56], another indication of the role of hedonicity in decision making.

In conclusion, the results support the idea of a dual process working of the mind. The elaboration likelihood model (ELM) is a model of how attitudes are formed and changed. Central to this model is the elaboration continuum, which ranges from low elaboration (low thought) to high elaboration (high thought) [57]. Central route processes are those that require a great deal of thought, involve careful scrutiny of the merits of arguments, and therefore are likely to predominate in rationality. Peripheral route processes, on the other hand, do not involve elaboration of the message and would predominate in hedonicity.

The result would support the conclusion that the main concern of decision makers is to maximize pleasure rather than rationality in their decisions. Even when told to make a rational choice, participants' decisions were close to their hedonic choices. In the "time pressure manipulation" in the first study, participants worked more under "system 1" type of thinking, and therefore make more "hedonic" choices. Although the a dual-process theory [7] has been challenged [58], the present results are compatible with Epstein's theory for decision making. Another way to present it would be motivational rationality *vs*. logical rationality [59]. The dual processes are rationality on the one hand and hedonicity on the other hand, with the later being the more fundamental one. When all of the necessary information is provided and enough time [26] is available, rationality and hedonicity tend to coincide, as they often do [60]. Actually, in many circumstances of life the hedonic choice is also the rational one. In such cases, there will be no difference between choices made under the two perspectives. When judging issues regarding different politics that are far from oneself (the war in Iraq, North Korea, the Palestinians in Israel, or Cuba), the decision makers make their judgments from a psychological distance. This perspective increases the likelihood that the decision maker will view the same alternative as rational and hedonic at the same time (since making a rational decision about political/social problem for other people, maximize one's pleasure, knowing "he did the right thing"). If such a mechanism takes place, it emphasizes the importance of hedonicity in the process of decision making.

Yet, hedonicity can lead to non-rational decisions and, consequently, its role is being recognized by more and more authors [47,48,61-63]. The acknowledgement of such a process in decision making has gained momentum in recent years as was recognized as the root of wanting [64], as the index of correct choice [65,66], as the anticipated source of reward in decision making [67], and especially in uncertain conditions when rationality is hindered [68]. "Pleasure has a central role in human life" [69]. Such a role is likely to be inherited from biology as hedonicity seems to be the decision maker in animals too [41].

## Competing interests

The author(s) declare that they have no competing interests.

## Authors' contributions

Both authors conceived the study, participated in its design carried out the sessions, drafted the manuscript, performed the statistical analysis, and read and approved the final manuscript.

## Supplementary Material

Additional file 1 Questionnaire 2. This questionnaire presents 10 items describing each five solutions to the social or political problem involved. Under each theme, indicate your choice on the attached 'response page.' Click here for file
