# Supplementary material for: Decision making: rational or hedonic?
Source: Behav Brain Funct. 2007 Sep 11;3:45. doi: 10.1186/1744-9081-3-45 (PMC2082033; doi:10.1186/1744-9081-3-45)
Supplement: Additional file 1 — Questionnaire 2. This questionnaire presents 10 items describing each five solutions to the social or political problem involved. Under each theme, indicate your choice on the attached 'response page.' [file 1744-9081-3-45-S1.rtf]

ADDENDUM

This questionnaire presents 10 items describing each five solutions to the social or political problem involved.  Under each theme, indicate your choice on the attached 'response page.'

1- War in Iraq
a- The Americans should leave Iraq and let the Iraqis solve their own problems.
b- The Americans should pacify Iraq completely before they leave the country.
c- UNO should replace the Americans and should send troops to pacify the country.
d- I am in favor of sending peace troops to help the population.
e- I am against sending troops, because that would be useless and young people will be killed.

2- Mundialization
a- I am in favor of free trade.  Thus, the poor countries will be able to participate in their development and to increase their wealth.
b- I am against free trade.  Thus, I protect my country's jobs.
c- I am against free trade because I believe that these treaties favor the big trusts at the expense of the poor populations.
d- I am in favor of free trade, but not through governments, especially those often corrupt in poor countries.
e- I am against total liberalization and support altermondialism.

3- Immigration
a- I believe that immigration should be completely free and that Canada should be a refuge territory.
b- I believe that to accept too many immigrants is a time bomb because those new citizens will not be able to assimilate, as they are too numerous.
c- I think that there should be a quota that should be revised each year.
d- I think that only people in good health should be accepted.
e- I believe that immigration is a good solution to our fall in birthrate.

4- Family
a- Low birth rate is a catastrophe. We should find a remedy as soon as possible.
b- Low birth rate is not a problem, there are enough living people on our planet.
c- Low birth rate is a very complex problem present in all developed countries. I don't see any solution to it.
d- Low birth rate is a very complex problem that we inherited from our parents with the idea that one should live for oneself.
e- The government should establish a coherent policy to help young families, with generous allocations, lower taxes with each child, and efficacious subventions to kindergartens.

5- Homosexuality
a- Homosexuals are normal people, they should have the same rights as other citizens.
b- Homosexuals are marginal people, although they pretend the contrary, they cannot have the same right to marriage as heterosexuals.
c- All discrimination must stop, homosexuals should be able to marry and adopt children.
d- Homosexuals should be able to marry but not to have children.
e- Homosexuals are only a minute minority in our population.  I am fed up with hearing of their supposed problems.

6- Abortion
a- Abortion is a crime that should be forbidden by law because the fetus cannot defend itself.
b- There are cases when abortion should be allowed, such as after rape or incest.
c- Abortion should be a problem for the woman only.
d- Decision to abort should be that of the physician and be controlled with a restrictive law.
e- To forbid abortion would take us 50 years back and the women who will want to abort will do it anyway at the risk of their health or their life.

7- GM foods
a- GM foods are a superb scientific advance.  They will allow more production at lower cost and thus will allow to feed more people in our time.
b- I am in favor to allow GM foods, they permit to reduce pesticides and thus are very ecological.
c- We are sorcerer's apprentices, we do not know how our body will react to these GM foods on the long term.
d- GM foods have always existed in nature. The present fear campaign is pushed by large agriculture producers because they fear that their income will drop.
e- Governments lie to us when they hide the dangers to consume GM foods. The law must inform consumers about what they buy.

8- North Korea and the atomic bomb
a- One should not trust the leaders of North Korea, it is urgent to inspect their nuclear progress, and it is a question of survival for us.
b- North Korea is a bloody dictatorship it is time to help its poor population.  Children die first in Korea.
c- We are not to start another war. The USA exaggerates the nuclear risk and the industrialized countries have nothing to fear from North Korea.
d- One should eliminate Kim Il Sung and all problems will be solved.
e- It is the North Korean's population problem, not ours.

9- Palestinians and Israelis
a- I am against any American help to the Israelis.
b- I am against any Arabian Countries help to the Palestinians.
c- That is a war between Jews and Moslems.  We are not concerned.
d- The Israelis crush the Palestinians.  It is Israel's fault if the Palestinians are so poor.  I am in favor of UNO intervention.
e- The Palestinians want to eliminate Israel by all means, including terrorism.  Israel defends itself that is all right.  I am in favor of Western Countries intervention.

10- Cuba is a totalitarian communist regime
a- That is not true, the citizens are quite happy in Cuba, I would like to live there.
b- That is true, and, those who can, flee Fidel Castro's regime.
c- That is not true, if the people are not happy, it the American blockade fault and not that of communism.
d- To say that prisons are full of political opponents is anti-communist propaganda.
e- Fidel Castro wants only his people's happiness, like Stalin...


NB.  The above presents Questionnaire 2.  The participants were invited to mark  in each of the 10 problems the solution they would decide to adopt.
Questionnaire 1, presented in random order the 50 entries (10 times a-e) to be rated hedonically.
